# Supplementary material for: HA-MOP knockin mice express the canonical µ-opioid receptor but lack detectable splice variants
Source: Commun Biol. 2021 Sep 14;4:1070. doi: 10.1038/s42003-021-02580-6 (PMC8440528; doi:10.1038/s42003-021-02580-6)
Supplement: Supplementary file 2 — Supplementary Information [file 42003_2021_2580_MOESM2_ESM.pdf]

HA-MOP knockin mice express the canonical μ-opioid receptor but lack detectable splice variants

Sebastian Fritzwanker[1], Lionel Moulédous[2], Catherine Mollereau[2,3], Carine Froment[3], Odile Burlet-Schiltz[3], Felix Effah[4], Alexis Bailey[4], Mariana Spetea[5], Rainer K. Reinscheid[1], Stefan Schulz[1]* and Andrea Kliewer[1]*

Supplementary Material and Methods

Chemicals

Radioligands [^{3}H][D-Ala^2,*N*-Me-Phe^4,Gly-ol^5]enkephalin ([^{3}H]DAMGO, 51.7 Ci/mmol) and [^{35}S]GTPγS (1250 Ci/mmol) were purchased from PerkinElmer (Boston, MA, USA). Guanosine diphosphate (GPD), GTPγS, DAMGO, tris(hydroxymethyl) aminomethane (Tris) and ethylene glycol-bis(β-aminoethyl ether)-*N*,*N*,*N'*,*N'*-tetraacetic acid (EGTA) were obtained from Sigma-Aldrich Chemicals (St. Louis, MO, USA). Morphine sulphate was obtained from Hameln Inc. (Hameln, Germany) and prepared as described in the main text. All other chemicals were of analytical grade and obtained from standard commercial sources.

Mouse brain membrane preparation

Membranes were prepared from wild-type (WT) and HA-MOP mouse brains as previously described[37]. Brains without cerebella were homogenized on ice in 5 volumes/weight of ice-cold 50 mM Tris-HCl buffer (pH 7.4) with a Teflon-glass homogenizer, and diluted in 30 volumes/weight of the same buffer. After centrifugation at 40,000 x *g* for 20 min at 4°C, pellets were resuspended in 30 volumes/weight of 50 mM Tris-HCl buffer (pH 7.4) and incubated at 37°C for 15 min. The centrifugation step described above was repeated, final pellets were resuspended in 5 volumes/weight of 50 mM Tris-HCl buffer (pH 7.4) containing 0.32 M sucrose and stored at -80°C until use. Prior to use in binding assays, membranes were thawed, washed

by centrifugation at 40,000 x *g* for 20 min at 4°C to remove sucrose. Protein concentration was determined by the method of Bradford using bovine serum albumin as standard[38].

[^{3}H]DAMGO saturation binding assay

Saturation binding experiments were performed in 50 mM Tris-HCl buffer (pH 7.4) in a final volume of 1 ml containing 0.2-0.3 mg protein as previously described[37]. Mouse brain membranes were incubated with different concentrations of [^{3}H]DAMGO (0.1-10 nM) at 25°C for 60 min. Non-specific binding was determined in the presence of 10 μM unlabelled DAMGO. Reactions were terminated by rapid filtration through Whatman glass GF/C fiber filters. Filters were washed three times with 5 ml of ice-cold 50 mM Tris-HCl buffer (pH 7.4) with a Brandel M24R cell harvester (Gaithersburg, MD). Radioactivity retained on the filters was counted by liquid scintillation counting using a Beckman Coulter LS6500 (Beckman Coulter Inc., Fullerton, CA). Saturation binding experiments were performed in duplicate and repeated five times.

[^{35}S]GTPγS binding assay

Mouse brain membranes (10 μg of protein) were incubated at 25°C for 60 min in Tris-EGTA buffer (50 mM Tris-HCl buffer, 3 mM $MgCl_2$, 1 mM EGTA, 100 nM NaCl, pH 7.4) containing 30 μM GDP, 0.05 nM [^{35}S]GTPγS and different concentrations of DAMGO (10^{-11}-10^{-5} M) in a final volume of 1 ml as previously described[37]. Non-specific binding was measured in the presence of 100 μM unlabelled GTPγS. Reactions were terminated by rapid filtration through Whatman GF/B glass fiber filters and bound [^{35}S]GTPγS retained on the filters was determined as described for the saturation binding assays. All experiments were performed in duplicate and repeated five times.

Data analysis

Nonlinear regression analysis of the saturation binding curves was performed to obtain the K_d (nM, equilibrium dissociation constant) and B_{max} (fmol/mg protein, receptor density) values

using GraphPad Prism software (GraphPad Prism Software Inc., San Diego, CA). Stimulation of [^{35}S]GTPγS binding produced by DAMGO is given as percentage of the basal activity (defined as 100%, measured in the absence of DAMGO). EC_{50} (nM, concentration of ligand to elicit half-maximal effect) and E_{max} (%, maximum stimulation) were calculated using nonlinear regression analysis with GraphPad Prism. Statistical analysis was performed using unpaired *t*-tests, and a P value < 0.05 was considered significant. Data are presented as means ± SEM.

MOP autoradiography in brains

General methods for autoradiographic binding were carried out as previously described by[33,34]. In brief, following decapitation, intact brains from WT and HA-MOP knockin male mice (10-12 weeks old) were removed, snap frozen at −20 °C in isopentane and stored at −80 °C. Brains were sectioned using a cryostat apparatus (Thermoscientific, UK) set at -21°C. Adjacent coronal brain sections, 20 μm thick, were cut at 300 μm intervals from fore to midbrain and thaw-mounted onto gelatine-coated ice-cold microscope slides for the determination of total and non-specific binding. Sections were rinsed for 30 min in a pre-incubation buffer solution (50 mM Tris-HCl, 0.9% w/v NaCl, pH 7.4 at room temperature). Total binding was determined by incubating the sections with [^{3}H]DAMGO (4 nM) in incubation buffer (50 mM Tris-HCl, pH 7.4 at room temperature) for 60 min. For non-specific binding, adjacent sections were incubated with 4 nM [^{3}H] DAMGO in the presence of 1 µM naloxone for 60 min. Slides were then rinsed three times for 5 min in ice-cold rinse buffer solution (50 mM Tris-HCl, pH 7.4 at 0ºC). Slides were then dried under a stream of cool air for 2 hours and stored in sealed containers with anhydrous calcium sulphate for 7 days. All slide were apposed to Kodak MR-1 films (Sigma-Aldrich) together with [^{3}H] microscales of known radioactive concentration for 7 weeks. Following development, autoradiographic films were analysed using MCID image analyzer (Imaging Research, Canada), as previously described by Kitchen and co-workers[35]. For each region quantified, measures were taken from both left and right hemispheres. The somatosensory and cingulate cortex was analysed by sampling 5–20 times with a box tool

(8 × 8 mm). All other regions were analysed by freehand drawing. Specific binding was calculated by subtraction of non-specific binding from total binding and expressed as fmol/mg tissue equivalents. Brain structures were identified by reference to the mouse atlas of Paxinos and Franklyn, (2001). Quantitative measures of radioligand binding were compared across genotype using one-way ANOVA (for the factors, genotype and region) followed by Bonferroni *post hoc* comparison if the ANOVA revealed significant ($P < 0.05$) effect. All statistical analyses were performed using GraphPad Prism 8.

Hot-plate test

Hot plate analgesia tests with WT and HA-MOP knockin male mice (10-12 weeks old) were performed as previously described in Kliewer et al. 2019[34]. Heterozygous MOP knockout mice ($MOP^{+/-}$) expressing only one copy of MOP were used for comparison (B6.129S2-*Oprm1*^{tm1Kff}/J, JAX™/Charles River Laboratories). In brief, effects on paw withdrawal latencies were assessed as the time to response (licking or flicking fore or hind paw(s)) after placement on a hot-plate maintained at 56°C (Ugo Basile SRL, IT). To avoid tissue damage, we used a 30-s cut-off was used. The hot-plate test was carried out 30 min after administration of cumulative morphine doses and antinociception is expressed as percent maximum possible effect (% MPE), calculated as follows: 100 × [(drug response latency − basal response latency)/(30 s − basal response latency)]. For consistency, the same experimenter performed the *in vivo* drug administrations and behavioural testing. All testing was conducted between 7 a.m. and 4 p.m. in an isolated, temperature- and light-controlled room. Mice were acclimated for at least 2 weeks before testing. Only the experimenter had free access to the room and entered the room 30 min before commencement of testing to eliminate potential olfactory-induced changes in nociception. Animals were assigned to groups randomly before testing. All experiments were randomized, performed by a blinded researcher, and then unblinded before statistical analysis. Antinociceptive responses to cumulative morphine doses were analyzed by two-way ANOVA

with “genotype” and “morphine dose” as variables, followed by Bonferroni’s post-hoc test wherever appropriate. A $P < 0.05$ was considered significant.

Supplementary Figure 1

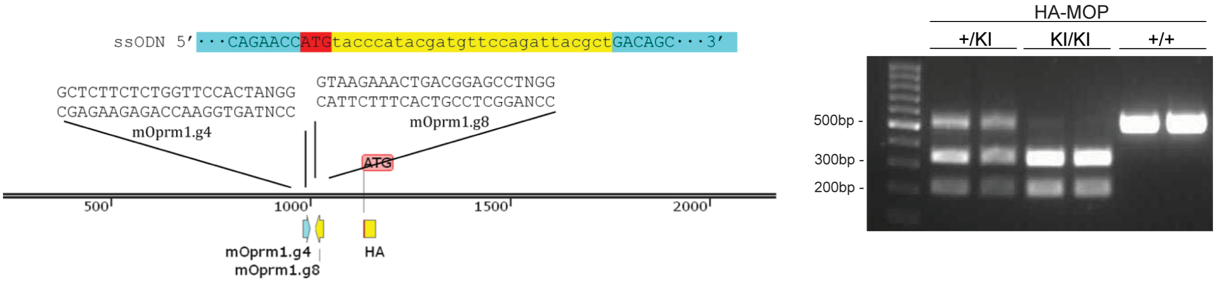

Suppl Fig 1: Design of HA-tagged mOprm1-knockin model. Insertion of the HA-tag into mouse Oprm1 (mOprm1) exon 1. Based on the validated guide RNA sequence, gRNA8, a single-strand oligo donor nucleotide (ssODN) was synthesized to insert an HA-tag downstream of the primary start of translation (ATG) of the mOprm1 gene (left panel). A 543bp PCR fragment generated by primer set mOprm 1F/1R will be present when the HA-tag is inserted in the mOprm1 gene. The WT allele (+) will give rise to a 516bp PCR fragment. In addition, an HA-tagged mOprm1 gene contains an RsaI restriction site in the PCR fragment, resulting in 208bp and 335bp fragments (right panel), while PCR products from the wild-type allele will not be cut. Legend: blue, genomic sequence; yellow, HA-tag; red, ATG start codon.

Supplementary Figure 2

	$[^3H]$DAMGO binding		$[^{35}S]$GTPγS binding	
	K_d (nM) ± SEM	B_{max} (fmol/mg protein) ± SEM	EC_{50} (nM) ± SEM	% stimulation ± SEM
WT	1.91 ± 0.23	46.5 ± 3.7	175 ± 23	153 ± 5
HA-MOP$^{KI/KI}$	2.04 ± 0.41	20.3 ± 1.8**	369 ± 81*	127 ± 3**

*P<0.05 and **P<0.01

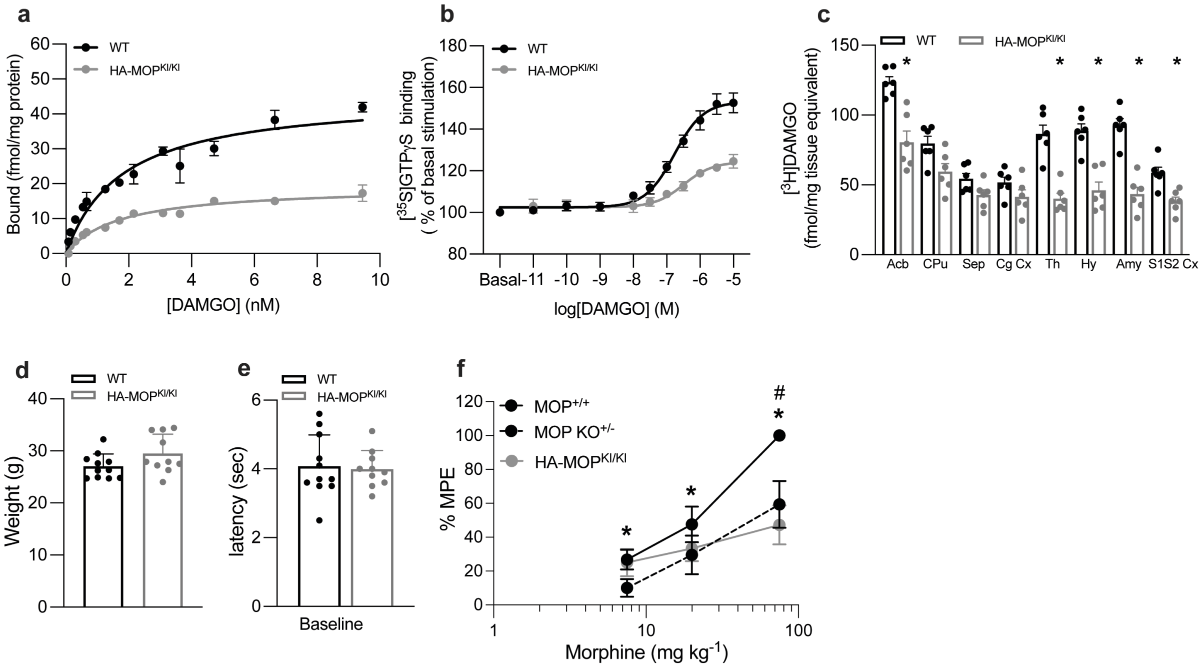

Suppl Fig 2: Pharmacological and physiological characterization of HA-MOP knockin mice. a Saturation binding of $[^3H]$DAMGO and **b** $[^{35}S]$GTPγS binding in WT and HA-MOP mouse brain membranes (n=5 per group), demonstrating a significant decrease in HA-MOP binding sites compared to WT-MOP. Data are presented as means ± SEM. Unpaired *t*-test, *P < 0.05, **P < 0.01. **c** Quantitative autoradiography of $[^3H]$DAMGO specific binding (4 nM) in brain sections from WT and HA-MOP mice. Data are expressed as means ± S.E.M (n=6 per brain area and genotype) specific $[^3H]$DAMGO binding (fmol/mg tissue equivalent). One-way ANOVA with Bonferroni *post hoc* test *(*P < 0.05*). Abbreviations: Acb, nucleus accumbens; CPu, caudate putamen; Sep, Septum; Cg Cx, cingulate cortex; Th, thalamus; Hy, hypothalamus; Amy, amygdala; S1S2 Cx, somatosensory cortex. **d** Bodyweight of HA-MOP mice compared to WT mice (aged 8-30 weeks, n= 10-11). **e** Basal nociceptive response measured in the mouse hot-plate test (n = 10-11). **f** Acute antinociceptive response measured in the mouse hot-plate test 30 min after administration of cumulative doses of morphine in wild-type (MOP KO$^{+/+}$), heterozygous MOP knockout (MOP KO$^{+/-}$) and HA-MOP mice (n = 4-

7). Nociceptive latencies were defined by paw withdrawals and are reported as percent maximum possible effect (% MPE) with a 30-s cut-off (genotype: $F_{(2, 12)} = 16.83$; *P = 0.003* and morphine dose: $F_{(2, 24)} = 1410$; *P < 0,0001*. Data are means ± s.e.m. * indicates statistically significant differences of MOP $KO^{+/-}$ compared to MOP $KO^{+/+}$; # indicates statistically significant differences of HA-$MOP^{KI/KI}$ compared to MOP $KO^{+/+}$; two-way ANOVA with Bonferroni *post-hoc* test.

Supplementary Table 1 Predicted molecular weights of MOP receptor splice variants. UniProtKB/Swiss-Prot identifiers are specified as accession number.

Isoform name	Accession number	Number of AAs	MW (Da)	MW with HA-tag* (Da)
MOP1	P42866-1	398	44421	45523
MOP1A	P42866-2	390	43563	44665
MOP1B1	P42866-3	391	43759	44861
MOP1B2	P42866-4	409	45911	47013
MOP1B3	P42866-5	392	43786	44888
MOP1B4	P42866-6	425	47752	48854
MOP1B5	P42866-7	388	43345	44447
MOP1C	P42866-8	438	48753	49855
MOP1D	P42866-9	393	43942	45044
MOP1E	P42866-10	401	44848	45950
MOP1F	P42866-11	444	49094	50196
MOP1O	P42866-12	416	46388	47490
MOP1P	P42866-13	453	50715	51817
MOP1U	P42866-17	474	52982	54084
MOP1V	P42866-18	420	47197	48299
MOP1W	P42866-19	399	44654	45756

* HA-tag: 1102 Da
