# Supplementary material for: HA-MOP knockin mice express the canonical µ-opioid receptor but lack detectable splice variants
Source: Commun Biol. 2021 Sep 14;4:1070. doi: 10.1038/s42003-021-02580-6 (PMC8440528; doi:10.1038/s42003-021-02580-6)
Supplement: Supplementary file 3 — Reporting summary [file 42003_2021_2580_MOESM3_ESM.pdf]

nature research

Corresponding author(s): Andrea Kliewer
Last updated by author(s): Aug 5, 2021

Reporting Summary

Nature Research wishes to improve the reproducibility of the work that we publish. This form provides structure for consistency and transparency in reporting. For further information on Nature Research policies, see our Editorial Policies and the Editorial Policy Checklist.

Statistics

For all statistical analyses, confirm that the following items are present in the figure legend, table legend, main text, or Methods section.

n/a	Confirmed	
☐	☒	The exact sample size (n) for each experimental group/condition, given as a discrete number and unit of measurement
☒	☐	A statement on whether measurements were taken from distinct samples or whether the same sample was measured repeatedly
☐	☒	The statistical test(s) used AND whether they are one- or two-sided<br>*Only common tests should be described solely by name; describe more complex techniques in the Methods section.*
☒	☐	A description of all covariates tested
☐	☒	A description of any assumptions or corrections, such as tests of normality and adjustment for multiple comparisons
☐	☒	A full description of the statistical parameters including central tendency (e.g. means) or other basic estimates (e.g. regression coefficient) AND variation (e.g. standard deviation) or associated estimates of uncertainty (e.g. confidence intervals)
☐	☒	For null hypothesis testing, the test statistic (e.g. F, t, r) with confidence intervals, effect sizes, degrees of freedom and P value noted<br>*Give P values as exact values whenever suitable.*
☒	☐	For Bayesian analysis, information on the choice of priors and Markov chain Monte Carlo settings
☒	☐	For hierarchical and complex designs, identification of the appropriate level for tests and full reporting of outcomes
☒	☐	Estimates of effect sizes (e.g. Cohen's d, Pearson's r), indicating how they were calculated

Our web collection on statistics for biologists contains articles on many of the points above.

Software and code

Policy information about availability of computer code

Data collection	Adobe: Illustrator, InDesign, Photoshop ; Microsoft Office
Data analysis	GraphPad Prism software

For manuscripts utilizing custom algorithms or software that are central to the research but not yet described in published literature, software must be made available to editors and reviewers. We strongly encourage code deposition in a community repository (e.g. GitHub). See the Nature Research guidelines for submitting code & software for further information.

Data

Policy information about availability of data

All manuscripts must include a data availability statement. This statement should provide the following information, where applicable:

- Accession codes, unique identifiers, or web links for publicly available datasets
- A list of figures that have associated raw data
- A description of any restrictions on data availability

The authors declare that all data supporting the findings of this study are available within the paper and its supplementary information files. The data that support the findings of this study are available from the authors upon reasonable request.

Field-specific reporting

Please select the one below that is the best fit for your research. If you are not sure, read the appropriate sections before making your selection.

☒ Life sciences ☐ Behavioural & social sciences ☐ Ecological, evolutionary & environmental sciences

For a reference copy of the document with all sections, see nature.com/documents/nr-reporting-summary-flat.pdf

Life sciences study design

All studies must disclose on these points even when the disclosure is negative.

Sample size	4-11
Data exclusions	no data were excluded
Replication	4-11
Randomization	Animals were assigned to groups randomly before testing.
Blinding	Animals were assigned to groups randomly before testing. All experiments were randomized, performed by a blinded researcher, and then unblinded before statistical analysis.

Reporting for specific materials, systems and methods

We require information from authors about some types of materials, experimental systems and methods used in many studies. Here, indicate whether each material, system or method listed is relevant to your study. If you are not sure if a list item applies to your research, read the appropriate section before selecting a response.

Materials & experimental systems

n/a	Involved in the study	
☐	☒	Antibodies
☐	☒	Eukaryotic cell lines
☒	☐	Palaeontology and archaeology
☐	☒	Animals and other organisms
☒	☐	Human research participants
☒	☐	Clinical data
☒	☐	Dual use research of concern

Methods

n/a	Involved in the study	
☒	☐	ChIP-seq
☒	☐	Flow cytometry
☒	☐	MRI-based neuroimaging

Antibodies

Antibodies used	Pierce™ Anti-HA Magnetic Beads were obtained from Thermo Fisher Scientific (Schwerte, Germany), while the phosphorylation-independent antibodies were obtained as follows: rabbit monoclonal anti-HA antibody (Cell Signaling, Frankfurt, Germany), and anti-MOP antibody {UMB-3} (Epitomics, Burlingame, CA); used as previously described17. The rabbit polyclonal phosphosite-specific µ-opioid receptor antibodies anti-pT370 (7TM0319B), anti-pT376 (7TM0319D) and anti-pT379 (7TM0319E) were obtained from 7TM Antibodies (Jena, Germany)18,19,20. The polyclonal phosphosite-specific anti-pS375 was obtained from Cell Signaling (Frankfurt, Germany). The polyclonal rabbit phosphorylation-independent-antibody for MOP1D was generated by S.S. the founder and scientific advisor of 7TM Antibodies GmbH, Jena, Germany against the alternative C-terminal splice sequence NHQRNEEPSS. This sequence corresponds to amino acids 384-393 of the postulated MOP1D mouse receptor. The antibodies were affinity-purified against their immunizing peptide using the SulfoLink kit (Thermo Scientific, Rockford, IL). In addition, the following commercially available secondary antibodies were used: polyclonal donkey anti-rabbit IgG Cy3 (Dianova, Hamburg, Germany) and goat anti-rabbit IgG, HRP-linked antibody (Cell Signaling, Frankfurt, Germany).
Validation	see webside

Eukaryotic cell lines

Policy information about cell lines

Cell line source(s)	HEK293 (human embryonic kidney 293 cells) cells were obtained from the German Resource Centre for Biological Material (DSMZ, Braunschweig, Germany)
Authentication	HEK293 (human embryonic kidney 293 cells) cells were obtained from the German Resource Centre for Biological Material (DSMZ, Braunschweig, Germany)

Mycoplasma contamination	no
Commonly misidentified lines (See ICLAC register)	*Name any commonly misidentified cell lines used in the study and provide a rationale for their use.*

Animals and other organisms

Policy information about studies involving animals; ARRIVE guidelines recommended for reporting animal research

Laboratory animals	Knock-in mice expressing HA-MOP (Oprm1em1Shlz, MGI:6117675) were generated by Applied StemCell (Menlo Park, USA), using CRISPR/Cas9-mediated targeted recombination
Wild animals	WT JAXTM C57BL/6J mice from Charles River Laboratories (DE) MGI:5000465)
Field-collected samples	non
Ethics oversight	All animal experiments were performed in accordance with relevant guidelines and regulations, were approved by Thuringian state authorities, and complied with European Commission regulations for the care and use of laboratory animals. Our study is reported in accordance with the ARRIVE (Animal Research: Reporting In Vivo Experiments) guidelines

Note that full information on the approval of the study protocol must also be provided in the manuscript.
